# 6-Fluoro-4-oxochroman-2-carboxylic acid

**DOI:** 10.1107/S1600536809054555

**Published:** 2010-01-09

**Authors:** Pei Chen, Shan Qian, Zhe-Qin Shi, Yong Wu

**Affiliations:** a Key Laboratory of Drug Targeting of the Education Ministry, West China School of Pharmacy, Sichuan University, Chengdu 610041, People’s Republic of China

## Abstract

The title compound, C_10_H_7_FO_4_, is an inter­mediate in the synthesis of the drug Fidarestat, (2*S*,4*S*)-2-aminoformyl-6-fluoro-spiro[chroman-4,4′-imidazolidine]-2′,5′-dione. The di­hydro­pyran­one ring adopts an envelope conformation with the asymmetric C atom in the flap position. In the crystal, the mol­ecules are linked into zigzag chains along [100] by O—H⋯O hydrogen bonds and C—H⋯π inter­actions involving the benzene ring.

## Related literature

Fidarestat, which shows strong inhibition to aldose reducta­ses, is used to treat complications of diabetes, see: Mealy (1996 ▶); Mitsuru *et al.* (2000 ▶). For related structures, see: Kurono *et al.* (1989 ▶); Yamaguchi *et al.* (1994 ▶).
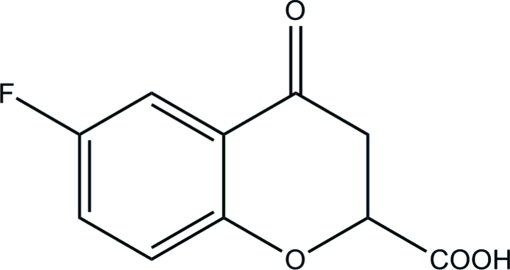


## Experimental

### 

#### Crystal data

C_10_H_7_FO_4_
*M* *_r_* = 210.16
Orthorhombic, 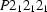

*a* = 5.3472 (11) Å
*b* = 12.748 (3) Å
*c* = 12.785 (3) Å
*V* = 871.5 (3) Å^3^
*Z* = 4
Mo *K*α radiation
μ = 0.14 mm^−1^
*T* = 113 K
0.28 × 0.23 × 0.22 mm

#### Data collection

Rigaku Saturn CCD area-detector diffractometer
Absorption correction: multi-scan (*ABSCOR*; Higashi, 1995 ▶) *T*_min_ = 0.962, *T*_max_ = 0.970
8640 measured reflections
1212 independent reflections
1049 reflections with *I* > 2σ(*I*)
*R*_int_ = 0.045

#### Refinement

*R*[*F* ^2^ > 2σ(*F* ^2^)] = 0.027
*wR*(*F* ^2^) = 0.073
*S* = 1.12
1212 reflections
137 parameters
H-atom parameters constrained
Δρ_max_ = 0.17 e Å^−3^
Δρ_min_ = −0.15 e Å^−3^

### 

Data collection: *CrystalClear* (Rigaku/MSC, 2005 ▶); cell refinement: *CrystalClear*; data reduction: *CrystalClear*; program(s) used to solve structure: *SHELXS97* (Sheldrick, 2008 ▶); program(s) used to refine structure: *SHELXL97* (Sheldrick, 2008 ▶); molecular graphics: *ORTEP-3* (Farrugia, 1997 ▶); software used to prepare material for publication: *SHELXL97*.

## Supplementary Material

Crystal structure: contains datablocks global, I. DOI: 10.1107/S1600536809054555/ci2987sup1.cif

Structure factors: contains datablocks I. DOI: 10.1107/S1600536809054555/ci2987Isup2.hkl

Additional supplementary materials:  crystallographic information; 3D view; checkCIF report

## Figures and Tables

**Table 1:** Hydrogen-bond geometry (Å, °)

*D*—H⋯*A*	*D*—H	H⋯*A*	*D*⋯*A*	*D*—H⋯*A*
O4—H4⋯O2^i^	0.84	1.81	2.6474 (19)	171
C5—H5*B*⋯*Cg*1^ii^	0.99	2.51	3.4521 (19)	160

Symmetry codes: (i) 
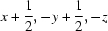
; (ii) 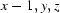. *Cg*1 is the centroid of the C1–C3/C7–C9 ring.

**Fig. 1.**
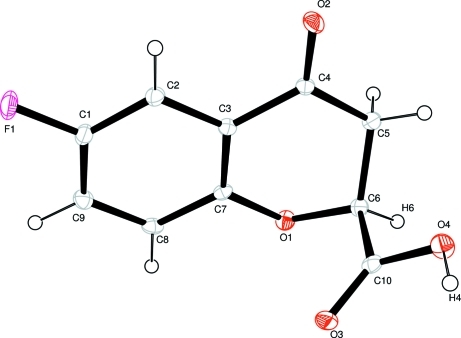
The molecular structure of the title compound, with displacement ellipsoids drawn at the 50% probability level.

## supplementary crystallographic information

### Comment

The optically active (*S*)-6-fluoro-4-oxochroman-2-carboxylic acid is a key intermediate for synthesizing Fidarestat which shows strong inhibition to aldose reductases to cure incurable complications of diabetes (Mitsuru *et al.*, 2000; Mealy, 1996). Our interests in synthesizing Fidarestat prompted us to develop an efficient methodology for synthesizing (*S*)-6-fluoro-4-oxochroman-2-carboxylic acid. In our synthetic work, we obtained the title compound, which is similar to those reported in the literature (Kurono *et al.*, 1989; Yamaguchi *et al.*, 1994). Its crystal structure is reported here.

The dihydropyranone ring adopts an envelope conformation with the asymmetric C atom in the flap position (Fig. 1). The molecules are linked into zigzag chains along the [100] by O—H···O hydrogen bonds and C—H···π interactions (Table 1) involving the benzene ring.

### Experimental

To a stirred solution of (2*S*,4*R*)-2-(1',2'-Dihydroxyethyl)-6-fluoro-chroman-4-one (10.7 g, 0.05 mol) in 300 ml of anhydrous benzene at room temperature was added lead tetraacetate (22.2 g, 0.05 mol). After 30 min, the solution was filtered and the filtrate was evaporated in vacuum to the residue. To the solution of 2% aqueous silver nitrate (651 ml, 0.07 mol) was added 5% aqueous sodium hydroxide (120 ml, 0.16 mol), and then generated the black precipitate immediately. To this stirred solution at room temperature was added, dropwise over 5 min, 4% ammonia water (520 ml, 0.16 mol). The black precipitate disappeared. The residue described above was dissolved in small amounts of THF and then added in this stirred solution at 323–333 K. After 10 min, the solution was filtered, and the precipitate was washed with water. The filtrate was acidified to pH 1 with 6 N aqueous hydrochloric acid, and then extracted with ethyl acetate. The organic extracts were dried (MgSO_4_) and then concentrated under reduced pressure. The residue was mixed with a mixture of ethanol and water, and left to crystallize 8.7 g (83%) of 6-Fluoro- 4-oxochroman-2-carboxylic acid. Colourless crystals suitable for X-ray analysis were obtained by slow evaporation in ethanol at room temperature.

### Refinement

H atoms were positioned geometrically (O-H = 0.84 and C-H = 0.95–1.00 Å) and refined using a riding model, with *U*_iso_(H) = 1.2*U*_eq_(C) and 1.5*U*_eq_(O). In the absence of significant anomalous scattering, Friedel pairs were merged prior to the final refinement. Nine reflections that were affected by the beamstop were discarded.

### Crystal data

**Table:**

C_10_H_7_FO_4_	*F*(000) = 432
*M**_r_* = 210.16	*D*_x_ = 1.602 Mg m^−^^3^
Orthorhombic, *P*2_1_2_1_2_1_	Mo *K*α radiation, λ = 0.71073 Å
Hall symbol: P 2ac 2ab	Cell parameters from 3184 reflections
*a* = 5.3472 (11) Å	θ = 1.6–27.8°
*b* = 12.748 (3) Å	µ = 0.14 mm^−^^1^
*c* = 12.785 (3) Å	*T* = 113 K
*V* = 871.5 (3) Å^3^	Block, colourless
*Z* = 4	0.28 × 0.23 × 0.22 mm

### Data collection

**Table:**

Rigaku Saturn CCD area-detector diffractometer	1212 independent reflections
Radiation source: rotating anode	1049 reflections with *I* > 2σ(*I*)
confocal	*R*_int_ = 0.045
Detector resolution: 7.31 pixels mm^-1^	θ_max_ = 27.9°, θ_min_ = 2.3°
ω and φ scans	*h* = −7→6
Absorption correction: multi-scan (*ABSCOR*; Higashi, 1995)	*k* = −16→16
*T*_min_ = 0.962, *T*_max_ = 0.970	*l* = −16→16
8640 measured reflections	

### Refinement

**Table:**

Refinement on *F*^2^	Primary atom site location: structure-invariant direct methods
Least-squares matrix: full	Secondary atom site location: difference Fourier map
*R*[*F*^2^ > 2σ(*F*^2^)] = 0.027	Hydrogen site location: inferred from neighbouring sites
*wR*(*F*^2^) = 0.073	H-atom parameters constrained
*S* = 1.12	*w* = 1/[σ^2^(*F*_o_^2^) + (0.0448*P*)^2^] where *P* = (*F*_o_^2^ + 2*F*_c_^2^)/3
1212 reflections	(Δ/σ)_max_ = 0.001
137 parameters	Δρ_max_ = 0.17 e Å^−^^3^
0 restraints	Δρ_min_ = −0.15 e Å^−^^3^

### Special details

**Table:**

Geometry. All e.s.d.'s (except the e.s.d. in the dihedral angle between two l.s. planes) are estimated using the full covariance matrix. The cell e.s.d.'s are taken into account individually in the estimation of e.s.d.'s in distances, angles and torsion angles; correlations between e.s.d.'s in cell parameters are only used when they are defined by crystal symmetry. An approximate (isotropic) treatment of cell e.s.d.'s is used for estimating e.s.d.'s involving l.s. planes.
Refinement. Refinement of *F*^2^ against ALL reflections. The weighted *R*-factor *wR* and goodness of fit *S* are based on *F*^2^, conventional *R*-factors *R* are based on *F*, with *F* set to zero for negative *F*^2^. The threshold expression of *F*^2^ > σ(*F*^2^) is used only for calculating *R*-factors(gt) *etc*. and is not relevant to the choice of reflections for refinement. *R*-factors based on *F*^2^ are statistically about twice as large as those based on *F*, and *R*- factors based on ALL data will be even larger.

### Fractional atomic coordinates and isotropic or equivalent isotropic displacement parameters (Å^2^)

**Table:**

	*x*	*y*	*z*	*U*_iso_*/*U*_eq_	
F1	0.8260 (2)	0.32440 (8)	0.43315 (9)	0.0336 (3)	
O1	0.2480 (2)	0.03466 (8)	0.23160 (9)	0.0189 (3)	
O2	0.1188 (2)	0.34646 (9)	0.17373 (10)	0.0230 (3)	
O3	0.5538 (2)	0.03536 (10)	0.06321 (10)	0.0230 (3)	
O4	0.2609 (3)	0.11076 (10)	−0.03716 (9)	0.0250 (3)	
H4	0.3799	0.1179	−0.0795	0.037*	
C1	0.6806 (3)	0.25426 (12)	0.38164 (13)	0.0200 (4)	
C2	0.5057 (3)	0.29048 (13)	0.31336 (12)	0.0184 (4)	
H2	0.4862	0.3636	0.3015	0.022*	
C3	0.3547 (3)	0.21751 (12)	0.26079 (12)	0.0152 (3)	
C4	0.1561 (3)	0.25266 (12)	0.19024 (12)	0.0154 (3)	
C5	−0.0026 (3)	0.16808 (13)	0.14292 (12)	0.0175 (3)	
H5A	−0.0588	0.1905	0.0726	0.021*	
H5B	−0.1529	0.1573	0.1868	0.021*	
C6	0.1404 (3)	0.06506 (12)	0.13377 (12)	0.0167 (3)	
H6	0.0182	0.0095	0.1130	0.020*	
C7	0.3894 (3)	0.10981 (12)	0.27966 (12)	0.0155 (4)	
C8	0.5700 (3)	0.07625 (13)	0.35030 (12)	0.0182 (4)	
H8	0.5923	0.0034	0.3630	0.022*	
C9	0.7161 (4)	0.14813 (12)	0.40169 (12)	0.0200 (4)	
H9	0.8396	0.1257	0.4502	0.024*	
C10	0.3450 (3)	0.06823 (12)	0.05065 (13)	0.0174 (3)	

### Atomic displacement parameters (Å^2^)

**Table:**

	*U*^11^	*U*^22^	*U*^33^	*U*^12^	*U*^13^	*U*^23^
F1	0.0418 (7)	0.0231 (5)	0.0358 (6)	−0.0017 (5)	−0.0248 (5)	−0.0041 (5)
O1	0.0218 (6)	0.0139 (5)	0.0211 (5)	−0.0031 (5)	−0.0003 (5)	0.0013 (4)
O2	0.0246 (7)	0.0158 (6)	0.0287 (6)	0.0020 (5)	−0.0091 (5)	0.0024 (5)
O3	0.0155 (6)	0.0265 (6)	0.0270 (6)	0.0037 (5)	0.0012 (5)	−0.0053 (5)
O4	0.0189 (6)	0.0340 (6)	0.0221 (6)	−0.0007 (6)	0.0050 (6)	0.0024 (5)
C1	0.0225 (10)	0.0191 (8)	0.0184 (7)	−0.0018 (7)	−0.0048 (7)	−0.0029 (7)
C2	0.0226 (10)	0.0146 (7)	0.0181 (7)	0.0007 (7)	−0.0026 (7)	−0.0004 (6)
C3	0.0155 (8)	0.0147 (7)	0.0155 (7)	0.0006 (6)	0.0004 (6)	0.0009 (6)
C4	0.0137 (8)	0.0165 (8)	0.0161 (7)	0.0007 (7)	0.0018 (6)	−0.0007 (6)
C5	0.0142 (8)	0.0182 (8)	0.0201 (8)	−0.0016 (7)	0.0011 (6)	−0.0024 (6)
C6	0.0151 (8)	0.0149 (7)	0.0201 (8)	−0.0027 (7)	0.0004 (6)	−0.0013 (6)
C7	0.0170 (9)	0.0140 (7)	0.0154 (7)	−0.0006 (7)	0.0045 (7)	−0.0012 (6)
C8	0.0226 (9)	0.0137 (7)	0.0181 (7)	0.0049 (7)	0.0033 (7)	0.0035 (6)
C9	0.0217 (9)	0.0229 (8)	0.0154 (7)	0.0042 (7)	−0.0006 (7)	0.0024 (6)
C10	0.0174 (9)	0.0133 (7)	0.0214 (8)	−0.0041 (7)	0.0010 (7)	−0.0048 (7)

### Geometric parameters (Å, °)

**Table:**

F1—C1	1.3557 (18)	C3—C4	1.464 (2)
O1—C7	1.366 (2)	C4—C5	1.499 (2)
O1—C6	1.4302 (19)	C5—C6	1.524 (2)
O2—C4	1.2305 (19)	C5—H5A	0.99
O3—C10	1.203 (2)	C5—H5B	0.99
O4—C10	1.325 (2)	C6—C10	1.526 (2)
O4—H4	0.84	C6—H6	1.00
C1—C2	1.360 (2)	C7—C8	1.390 (2)
C1—C9	1.390 (2)	C8—C9	1.372 (2)
C2—C3	1.403 (2)	C8—H8	0.95
C2—H2	0.95	C9—H9	0.95
C3—C7	1.406 (2)		
			
C7—O1—C6	115.20 (12)	H5A—C5—H5B	108.0
C10—O4—H4	109.5	O1—C6—C5	111.60 (12)
F1—C1—C2	118.84 (14)	O1—C6—C10	109.14 (14)
F1—C1—C9	118.29 (15)	C5—C6—C10	112.98 (13)
C2—C1—C9	122.87 (16)	O1—C6—H6	107.6
C1—C2—C3	118.56 (15)	C5—C6—H6	107.6
C1—C2—H2	120.7	C10—C6—H6	107.6
C3—C2—H2	120.7	O1—C7—C8	117.44 (14)
C2—C3—C7	119.28 (15)	O1—C7—C3	122.30 (15)
C2—C3—C4	120.64 (14)	C8—C7—C3	120.25 (15)
C7—C3—C4	120.03 (14)	C9—C8—C7	120.09 (15)
O2—C4—C3	121.39 (14)	C9—C8—H8	120.0
O2—C4—C5	122.54 (15)	C7—C8—H8	120.0
C3—C4—C5	116.04 (13)	C8—C9—C1	118.95 (16)
C4—C5—C6	111.51 (13)	C8—C9—H9	120.5
C4—C5—H5A	109.3	C1—C9—H9	120.5
C6—C5—H5A	109.3	O3—C10—O4	124.77 (16)
C4—C5—H5B	109.3	O3—C10—C6	124.27 (15)
C6—C5—H5B	109.3	O4—C10—C6	110.96 (14)
			
F1—C1—C2—C3	−179.60 (15)	C6—O1—C7—C3	24.6 (2)
C9—C1—C2—C3	−0.1 (3)	C2—C3—C7—O1	179.64 (14)
C1—C2—C3—C7	−0.4 (2)	C4—C3—C7—O1	2.1 (2)
C1—C2—C3—C4	177.15 (14)	C2—C3—C7—C8	0.5 (2)
C2—C3—C4—O2	1.2 (2)	C4—C3—C7—C8	−177.00 (14)
C7—C3—C4—O2	178.73 (16)	O1—C7—C8—C9	−179.41 (14)
C2—C3—C4—C5	−176.75 (14)	C3—C7—C8—C9	−0.3 (2)
C7—C3—C4—C5	0.8 (2)	C7—C8—C9—C1	−0.2 (2)
O2—C4—C5—C6	154.54 (16)	F1—C1—C9—C8	179.88 (15)
C3—C4—C5—C6	−27.50 (19)	C2—C1—C9—C8	0.3 (3)
C7—O1—C6—C5	−52.00 (18)	O1—C6—C10—O3	10.0 (2)
C7—O1—C6—C10	73.59 (16)	C5—C6—C10—O3	134.74 (16)
C4—C5—C6—O1	53.01 (17)	O1—C6—C10—O4	−170.78 (12)
C4—C5—C6—C10	−70.43 (17)	C5—C6—C10—O4	−46.00 (17)
C6—O1—C7—C8	−156.25 (14)		

### Hydrogen-bond geometry (Å, °)

**Table:**

*D*—H···*A*	*D*—H	H···*A*	*D*···*A*	*D*—H···*A*
O4—H4···O2^i^	0.84	1.81	2.6474 (19)	171
C5—H5B···Cg1^ii^	0.99	2.51	3.4521 (19)	160

Symmetry codes: (i) *x*+1/2, −*y*+1/2, −*z*; (ii) *x*−1, *y*, *z*.
